# Dimensional Engineering of 1D/2D Synergistic TiO_2_ Nanostructures for High-Efficiency Photocatalytic CO_2_ Reduction

**DOI:** 10.3390/ma18174148

**Published:** 2025-09-04

**Authors:** Xiang Liu, Fujiang Huang, Xiang Shi, Hangmin Xu, Jian Xu, Xingwang Zhu

**Affiliations:** Institute of Technology for Carbon Neutralization, School of Environmental Science and Engineering, Yangzhou University, Yangzhou 225009, China

**Keywords:** photocatalysis, CO_2_ reduction, TiO_2_, morphological structure

## Abstract

Alongside the gradual progress of industrialization and the continuous development of human society, the problems of environmental pollution and energy crisis have become increasingly prominent. Semiconductor photocatalysis is a promising solution to these challenges. The photocatalytic reduction of CO_2_ by TiO_2_ to produce carbon monoxide and methane is a process which has been identified as a means of developing clean energy. In this paper, two-dimensional TiO_2_ (2D-TiO_2_) was synthesized via a one-step solvothermal method, and one-dimensional TiO_2_ (1D-TiO_2_) was obtained through a hydrothermal process. Their photocatalytic CO_2_ reduction performances were systematically investigated. The results show that 2D-TiO_2_ exhibits superior catalytic activity compared to 1D-TiO_2_, which can be attributed to its lamellar structure, larger specific surface area, and improved hydrophilicity, providing more active sites and faster reaction kinetics. To further reveal the reaction mechanism, density functional theory (DFT) calculations were carried out using VASP with the GGA–PBE functional, PAW potentials, and a plane-wave cutoff energy of 520 eV. A 3 × 3 × 1 Monkhorst–Pack grid was used for Brillouin zone integration, and all possible adsorption configurations of CO_2_*, COOH*, and CO* intermediates on the 2D-TiO_2_ surface were evaluated. The results confirm that 2D-TiO_2_ stabilizes key intermediates more effectively, thereby lowering the energy barrier and facilitating CO_2_ reduction. These findings demonstrate that structural modulation of TiO_2_ significantly influences its photocatalytic performance and highlight the great potential of 2D-TiO_2_ for efficient CO_2_ conversion and clean energy applications.

## 1. Introduction

In recent years, the excessive emission of greenhouse gases (GHGs) has triggered the serious challenge of global warming. The efficient conversion and utilization of carbon dioxide (CO_2_), as one of the major GHGs, has become a research hotspot in the field of energy and the environment [1,2]. The transformation of CO_2_ into valuable fuels and chemicals is regarded as an important solution to solve the energy crisis. In recent years, researchers have attempted to convert CO_2_ into energy and chemicals through methods such as electrocatalysis, thermocatalysis, and photocatalysis to promote sustainable development and achieve carbon neutrality, with photocatalysis serving as a key bridge to achieve this goal [3,4,5,6,7].

Two-dimensional (2D) materials have a unique lamellar planar structure, with lateral dimensions that can easily exceed 100 nm, or even reach the range of a few micrometers, while their thickness can be accurately controlled to a single cellular atomic layer or just a few cellular atomic layers. This special atomic structure and the high specific surface area, extreme thinness, and the remarkable quantum confinement effect experienced by electrons in two dimensions combine to give 2D materials extraordinary physical and chemical properties. Various surface modification methods have been investigated to enhance the effectiveness of these materials as photocatalysts [8]. Relatively one-dimensional (1D) nanostructures cover rod, wire, tube, ribbon, hook, needle and zigzag structures. These structures are characterized by the fact that one of the dimensions is significantly larger than the other, and this size difference is equivalent to the nanoscale (length versus width or diameter). In many literature reports, they are often presented in the form of needle-like structures, which can extend even to a millimeter scale in length. Comparatively, the flake form is still more likely to react in contact with CO_2_.

The large specific surface area and ultra-thin thickness of two-dimensional structures provide more surface active sites and shorten the transport path of photo-generated carriers, thereby improving charge separation efficiency and utilization [9]. It is evident that two-dimensional nanosheets have attracted considerable attention in the field of photocatalysis research due to their distinctive properties. At same time, previous studies have employed bespoke density functional theory (DFT) methodologies to methodically investigate semiconductor configurations and heterostructures exhibiting one-dimensional and two-dimensional characteristics. These investigations have elucidated the intricate interplay between their electronic structures and photocatalytic properties [10,11]. Present studies provide a theoretical basis for understanding the relationship between 1D/2D characteristics and semiconductor performance, and they also provide important references for the TiO_2_ two-dimensional system that is the focus of this work.

TiO_2_ is a classical photocatalytic semiconductor material and is widely used for pollutant degradation, photocatalytic CO_2_ reduction, water decomposition, and solar cells. Initially, most of the studies focused on 0D TiO_2_ nanoparticles, which have excellent catalytic activity due to their excellent chemical stability and wide bandgap (~3.2 eV), enabling efficient excitation of electron–hole pair separation. However, the limited utilization of visible light leads to low solar photovoltaic efficiency and easy complexation of photogenerated electron–hole pairs [12]. In recent years, 2D-TiO_2_ nanostructures have attracted considerable attention due to their unique advantages. Typically, 2D-TiO_2_ exists in the form of ultra-thin nanosheets or layered structures, featuring a high specific surface area that provides abundant CO_2_ adsorption sites and reaction intermediate anchoring points. This enhances the efficiency of photocatalytic reactions [13]. In addition, the 2D-TiO_2_ layer is extremely thin, thereby ensuring a minimal path length for photogenerated electrons to migrate from the excitation point to the surface. This property effectively inhibits electron–hole complexation. Therefore, noble metal loading [14], doping with non-metallic elements [15], structural modulation, and defect engineering [16] have been used to solve the problems. In these strategies, morphological and structural adjustments are considered effective and promising approaches, given the potential for promoting light-induced electron–hole pair separation. The augmented specific surface area of nanosheets has been demonstrated to increase the number of active sites on the surface, thereby enhancing charge transfer and the efficiency of light-excited electron–hole pair separation [17]. It is noteworthy that extant research suggests that the electronic structure of TiO_2_ is predominantly determined by atomic configuration as opposed to particle size. For example, Cho et al. performed density functional theory (DFT)/time-dependent density functional theory (TD-DFT) calculations on TiO_2_ nanoparticles ranging from 0.5 to 3.2 nm in size and found that the changes in electronic structure were primarily due to different isomers rather than size differences [18]. Moreover, first-principles studies of TiO_2_ defects also indicate that even when the supercell size changes, its main electronic structure characteristics remain consistent [19]. Therefore, the development of two-dimensional semiconductor photocatalysts with elevated specific surface areas represents a highly promising research direction. However, given the established status of TiO_2_ as a conventional photocatalyst, there is a paucity of literature concerning the preparation of two-dimensional sheet structures of TiO_2_.

In this work, 2D-TiO_2_ catalysts were prepared and synthesized by a one-step solvothermal method, and 1D titanium oxide (1D-TiO_2_) was also synthesized by a hydrothermal method. Meanwhile, the photocatalytic reduction of CO_2_ by 2D-TiO_2_ exhibited superior photocatalytic performance, which was more than twice as effective as that of 1D-TiO_2_. Their CO yields were 21.53 μmol g^−1^ h^−1^ and 14.69 μmol g^−1^ h^−1^, respectively. Through this work, it is possible to provide insights into the unique role of shape dimension modulation in CO_2_ adsorption and activation.

## 2. Experiments

### 2.1. Chemical Reagents

Tetrabutyl titanate (C_16_H_36_O_4_Ti, analytically pure), titanium trichloride (TiCl_3_, analytically pure) were purchased from Aladdin Reagent Inc. (Shanghai, China). HNO_3_ (analytically pure), hydrofluoric acid (HF, analytically pure), triethanolamine (TEOA, analytically pure), acetonitrile (MeCN, analytically pure), ethanol (EtOH, analytically pure) were purchased from Sinopharm Chemical Reagent Co. (Shanghai, China).

### 2.2. Catalyst Synthesis

#### 2.2.1. Preparation of 2D-TiO_2_

In a magnetic stirring device, 1.6 mL of HF was added dropwise to 10 mL of C_16_H_36_O_4_Ti solution. The mixture was subjected to continuous stirring for a period of 10 min, after which it was transferred to a reactor vessel equipped with a polytetrafluoroethylene liner. Subsequently, the reactor was kept at 200 °C for 24 h. At the end of the experiment, the reactor was permitted to undergo natural cooling processes until it had reached ambient temperature. Thereafter, the resulting products were subjected to a series of washing operations using deionized water and anhydrous ethanol, respectively, with the addition of water bath ultrasonication for a duration of two hours. The final product was found to be a flaky 2D-TiO_2_.

#### 2.2.2. Preparation of 1D-TiO_2_

Titanium trichloride (TiCl_3_) with a volume ratio of 1:5 (TiCl_3_:H_2_O) was added to deionized water at room temperature and stirred continuously for 5 min. Following this, the solution was fully mixed and homogeneous. It was then transferred to a 100 mL reactor and placed in a blast drying oven for heating treatment. The solution was allowed to react for 24 h under the condition that the temperature of the blast drying oven was adjusted to 180 °C. At the end of the reaction, the product was allowed to cool naturally and slowly to room temperature in the drying oven. After removing the supernatant, the product was washed three times with deionized water and anhydrous ethanol to remove impurities, yielding rod-like TiO_2_ nanofluids.

### 2.3. Activity Test

The performance test of the photocatalytic reduction of CO_2_ was conducted using a series of catalysts, including triethanolamine, acetonitrile, and deionized water. Firstly, the reactor was washed clean, and then 10 mg of the sample was added into the reactor containing 6 mL of MeCN, 4 mL of deionized water, and 2 mL of TEOA (MeCN:H_2_O:TEOA = 3:2:1) and shaken in an ultrasonic cleaner to form a homogeneous solution. The reactor was then evacuated, followed by purging the system with high-purity CO_2_ gas to remove excess air impurities, and then evacuated again to remove excess gas remaining in the recirculation system. After completing the above operations, high-purity CO_2_ was reintroduced into the circulatory system, and the system pressure was adjusted to about 80 kPa to start the experiment. During the experiment, the catalyst in the reactor was cooled by condensate at 10 °C and illuminated by a 300 W xenon lamp. At hourly intervals, 1 mL of reaction gas was extracted from the system and analyzed by gas chromatography.

## 3. Results and Discussion

**Figure 1:**
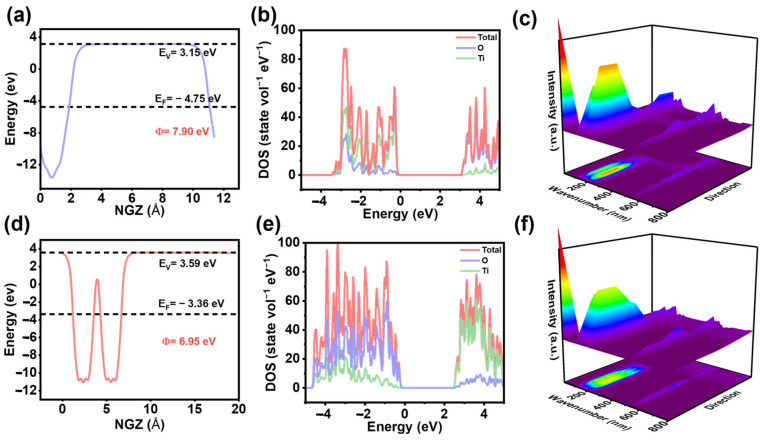
Electrostatic potential calculation along the z-axis of (**a**) 1D-TiO_2_ and (**d**) 2D-TiO_2_. DOS of (**b**) 1D-TiO_2_ and (**e**) 2D-TiO_2_. Light absorption spectra of (**c**) 1D-TiO_2_ and (**f**) 2D-TiO_2_.

To confirm the hypothesis that 2D-TiO_2_ improves CO_2_ reduction, computational models of 1D-TiO_2_ and 2D-TiO_2_ were constructed (Figure S1). The effect of morphology on the electronic configuration and band structure of TiO_2_ was revealed through calculations employing density functional theory (DFT). The work functions (WFs) of 1D-TiO_2_ and 2D TiO_2_ were calculated (Figure 1a,d). The WF of 1D-TiO_2_ is 6.95 eV, whereas the WF of 2D-TiO_2_ is 7.90 eV, and the difference is primarily attributed to the morphology tuning of TiO_2_, which increases the WF of 2D-TiO_2_. Furthermore, alterations in the crystal structure have the capacity to influence the photogenerated carrier transfer behaviors and band structures [20]. As illustrated in Figure 1b,e, 2D-TiO_2_ demonstrates a superior density of states (DOS) at the Fermi level in comparison to 1D-TiO_2_, which promotes photogenerated electron–hole pair transfer, and the bandgap of the 2D-TiO_2_ band structure is reduced, as shown in Figure S2 [21]. This phenomenon is indicative of the material’s augmented capacity for light absorption. To prove the above conclusions, the light absorption spectra of the materials were calculated. As shown in Figure 1c,f, the change in morphology leads to enhanced absorption of light by the catalysts [22].

**Figure 2:**
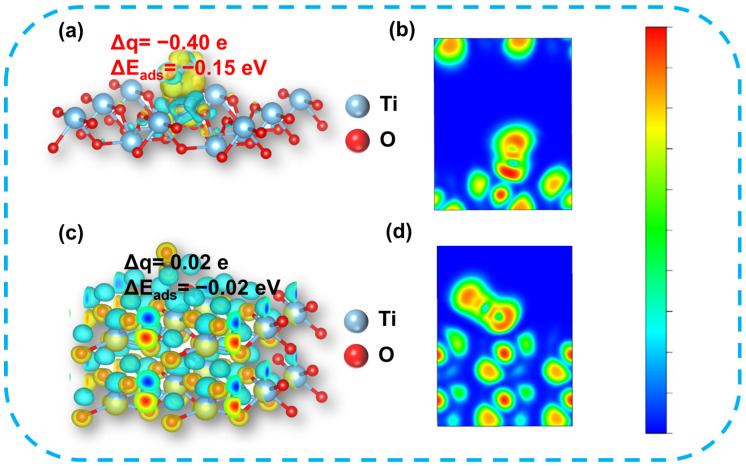
Charge density difference of CO_2_ adsorbed on (**a**) 2D-TiO_2_ and (**c**) 1D-TiO_2_ (Δq represents the total charge of CO_2_) and ELF of CO_2_ adsorbed on (**b**) 2D-TiO_2_ and (**d**) 1D-TiO_2_.

The electron transport capability of the materials was investigated using the electron localization function (ELF). The adsorption capacity of CO_2_ on the 2D-TiO_2_ was investigated by difference charge analysis. The results showed that the adsorption energy of 2D-TiO_2_ for CO_2_ was reduced by 0.13 eV compared to 1D-TiO_2_ (Figure 2a). This reduction suggests that the exposed surface of 2D-TiO_2_ can provide additional active sites for the adsorption and activation of CO_2_ molecules, thus promoting their adsorption [23,24]. Additionally, 2D-TiO_2_ (−0.40 e) has a higher electron density than 1D-TiO_2_ (0.02 e), allowing for more electrons to be transferred to CO_2_ and making C–O bond dissociation easier. As shown in Figure 2a,c, the accumulation and depletion of electrons were observed, with the yellow areas representing the accumulation of aggregated electrons and the blue areas representing the depletion of transferred electrons [25]. The results indicate that the greater surface area of 2D-TiO_2_ leads to an increased enrichment of electrons and CO_2_ on the catalyst surface, and the thinner thickness is more conducive to the transfer of electrons. Furthermore, Figure 2c,d demonstrate that the surface charge density of 2D-TiO_2_ exceeds that of 1D-TiO_2_, enabling a greater number of electrons to escape from the surface, thereby facilitating the adsorption and activation of CO_2_. The tuning of the TiO_2_ morphology increased the active sites of the catalyst, thereby increasing the charge density of the catalyst. Theoretical calculations showed that the change in morphology could improve photocatalytic capacity.

The crystallographic structure and phase purity of the as-synthesized TiO_2_ samples were examined by XRD analysis. As shown in Figure S3, the XRD pattern of 1D shows several diffraction peaks, which are strong and sharp, indicating that 1D is highly crystalline [26]. 2D-TiO_2_ and 1D-TiO_2_ have several peaks, observed at 2θ = 27.45°, 36.09°, 39.19°, 41.23°, 54.32°, 62.74°, and 64.04° and all of them correspond to the peaks of the catalyst. The diffraction peaks of the standard card JCPDS No. 21-1276 confirmed the successful synthesis of TiO_2_. The 2θ = 27.45° corresponds to the (110) face of rutile titanium dioxide, and it can also be seen from the figure that the synthesized products are all pure phases of the rutile phase, and there are no other impurity diffraction peaks.

The composition of the material was determined by means of Fourier transform infrared (FT-IR) spectroscopy, with the functional groups of the sample (as illustrated in Figure S4) being utilized for this purpose. It can be observed that the FT-IR curve of 2D-TiO_2_ is almost identical to that of 1D-TiO_2_, but the peaks of 2D-TiO_2_ are strong and sharp. The absorption peaks of the 2D-TiO_2_ curve appear at 1631–1648 cm^−1^, which is attributed to the presence of carboxyl groups. The broad band between 3400 and 2300 cm^−1^ in the FT-IR spectrum is attributed to the O-H stretching mode of the adsorbent. Meanwhile, the peak at 2928 cm^−1^ corresponds to the C-H stretching vibration of the sample, and the weak band located at 1624 cm^−1^ is related to the liganded H_2_O and Ti-OH bending vibrations.

**Figure 3:**
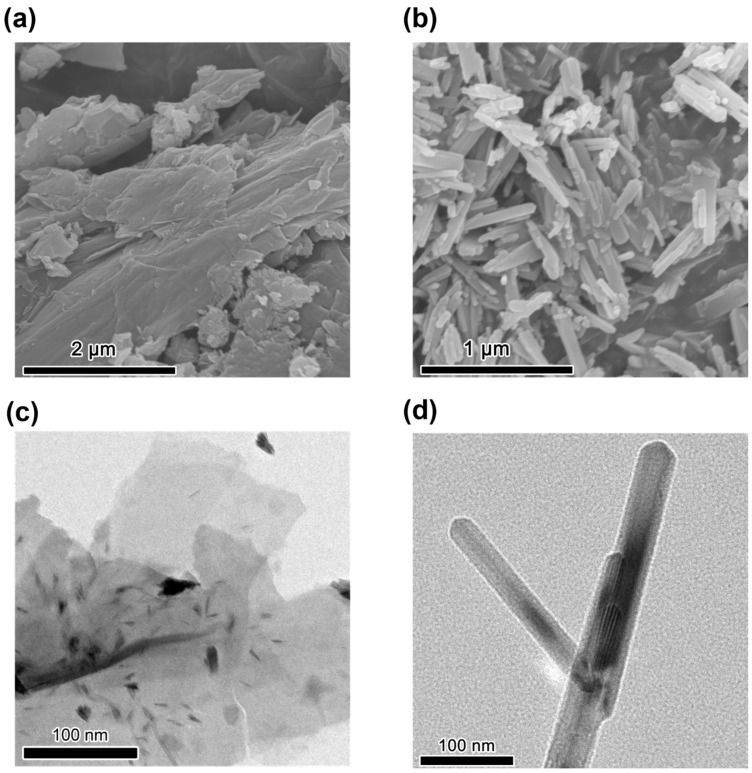
Morphology characterization of 2D-TiO_2_ and 1D-TiO_2_. (**a**) SEM images of (**a**) 2D-TiO_2_ and (**b**) 1D-TiO_2_. HRTEM images of (**c**) 2D-TiO_2_ and (**d**) 1D-TiO_2_.

To confirm the morphology and structure of 2D-TiO_2_ and 1D-TiO_2_, the morphology was characterized by scanning electron microscopy (SEM) and high-resolution transmission electron microscopy (HRTEM). As demonstrated in Figure 3a, 2D-TiO_2_ has a sheet-like structure. As demonstrated in Figure 3b, 1D-TiO_2_ exhibits a rod-like structure, with nanorods that are closely spaced with no interstitial gaps between the sidewalls. This suggests that the roughness of 1D-TiO_2_ is minimal. The 2D-TiO_2_ flakes exhibit an augmented surface area, facilitating enhanced interaction with CO_2_. Their thickness is notably thinner compared to 1D-TiO_2_, thereby enhancing the efficiency of electron transport. This enhancement of the diffusion performance of reactants within the catalyst is known to promote the mass transfer process, thereby reducing mass transfer resistance and improving the reaction rate and selectivity. Figure 3c,d illustrate images of 2D-TiO_2_ and 1D-TiO_2_, respectively, as observed under a high-resolution transmission electron microscope. These images are consistent with the results obtained from the SEM analysis, thereby providing further confirmation of the flake and rod structures. The small dots visible in the shadows of the flake structures may be attributable to Ti-O clusters on the surface of the crystals [27].

An analysis was conducted on the structure and morphology of the materials to ascertain the feasibility of their photocatalytic reduction of CO_2_. The present study investigates the charge difference and adsorption energy of CO_2_ on 1D-TiO_2_ and 2D-TiO_2_ using simulations. It can be seen that from the difference in charge density between 1D-TiO_2_ and 2D-TiO_2_, the CO_2_ adsorption energy of 2D-TiO_2_ (−0.15 eV) is significantly lower than that of 1D-TiO_2_ (−0.02 eV), indicating that 2D-TiO_2_ is more favorable for CO_2_ adsorption [28,29], thus facilitating electron transfer to CO_2_ and enhancing the dissociation of the C-O bond. Furthermore, 2D-TiO_2_ has been shown to possess a greater number of active sites, resulting in an increased number of electrons available for CO_2_ activation due to an expanded surface area [30,31]. Theoretical analyses of charge difference and ELF demonstrated that the CO_2_ reduction performance of 2D-TiO_2_ was significantly enhanced in comparison with that of 1D-TiO_2_.

**Figure 4:**
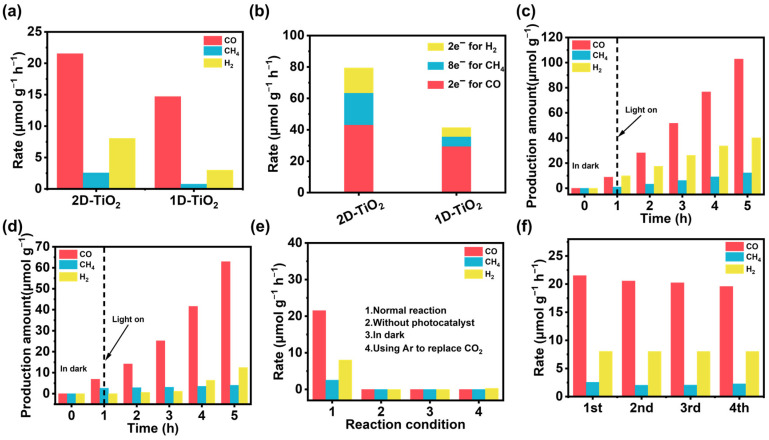
(**a**) Photocatalytic CO_2_ reduction performance of 2D-TiO_2_ and 1D-TiO_2_. (**b**) Comparison of molar electron numbers for CO_2_ reduction processes. CO_2_ reduction over time of (**c**) 2D-TiO_2_ and (**d**) 1D-TiO_2_. (**e**) Cyclic experiment of 2D-TiO_2_ sample. (**f**) Conditional experimental analysis.

An investigation into the performance of the samples in the CO_2_ photocatalytic reduction reaction was conducted through a comprehensive analysis of their composition and structure. As shown in Figure 4a, the CO yield of 1D-TiO_2_ was as high as 14.69 μmol g^−1^ h^−1^, and the CH_4_ yield was 0.77 μmol g^−1^ h^−1^, whereas the morphology of TiO_2_ was altered. The CO and CH_4_ yields were 21.53 μmol g^−1^ h^−1^ and 2.55 μmol g^−1^ h^−1^, and the CO activity was increased by 1.5-fold and the CH_4_ activity by 10-fold. The superiority of this performance was evaluated by comparing it with a representative commercial P25 TiO_2_ reported in the literature [32]. In contrast, the catalyst prepared in this study demonstrated significantly improved performance, with the yield of CO increasing by around 9-fold (Figure S5). In addition, the moles of photoelectrons involved in the reduction of CO_2_ to produce CO and CH_4_ were calculated for 1D-TiO_2_ and 2D-TiO_2_. As shown in Figure 4b, 2D-TiO_2_ has a higher electron ratio when reducing CO_2_ to CH_4_, and more electrons are involved in the CO_2_ reduction reaction. This shows that 2D-TiO_2_ has the potential to produce high value-added products. Next, the activities of 2D-TiO_2_ and 1D-TiO_2_ were investigated under continuous dark to light conditions as well as in continuous experiments. As shown in Figure 4c,d, CO and CH_4_ were not produced in the absence of light, and their production increased rapidly after light exposure [33]. The conditioned experiments are shown in Figure 4f. Furthermore, the absence of a photocatalyst results in an absence of a product reduction, thereby indicating that the entirety of the photocatalytic reaction process necessitates both a light source and a photocatalyst. In the experiment, the reaction atmosphere was altered from CO_2_ to Ar. This resulted in no CO or CH_4_ being detected, and a small amount of hydrogen was produced. This finding indicates that the carbon in the products originated from the CO_2_ in the reaction, and that a certain amount of water decomposition occurred within the system, as shown in Figure 4e. Finally, the photocatalytic performance of the 2D-TiO_2_ catalyst for CO_2_ reduction exhibited a slight decrease after four consecutive cycles (20 h) of activity testing. In summary, the 2D-TiO_2_ catalyst demonstrated satisfactory stability in the context of photocatalytic CO_2_ reduction. XRD analysis before and after the reaction (Figure S6) showed no change in the peak position, but a slight decrease in the peak intensity, indicating a decrease in crystallinity.

**Figure 5:**
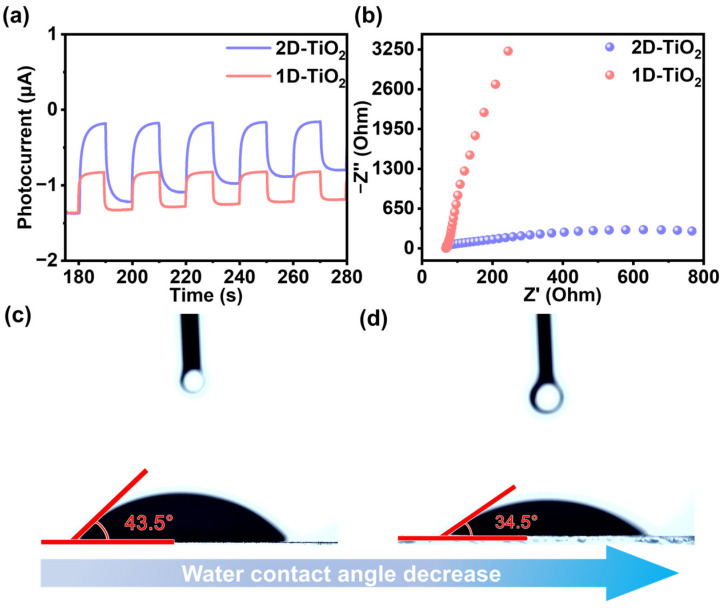
(**a**) Photocurrent response analysis, and (**b**) EIS of 2D-TiO_2_ and 1D-TiO_2_. Water contact angles of (**c**) 1D-TiO_2_ and (**d**) 2D-TiO_2_.

In the area of photocatalysis, photocurrent measurements are commonly used to evaluate the effectiveness of photogenerated electron–hole pair separation. As shown in Figure 5a, such measurements were performed on 1D-TiO_2_ and 2D-TiO_2_ catalysts. The results reveal that 2D-TiO_2_ exhibits a higher instantaneous photocurrent compared to its 1D-TiO_2_. This finding suggests that the 2D-TiO_2_ catalyst possesses significant advantages in the transfer and separation of photogenerated carriers [34,35]. The photogenerated electron–hole pairs are more easily separated, which further indicates that the photogenerated carriers are transported more rapidly on 2D-TiO_2_ [36]. This is also evidenced by the lateral separation of photogenerated charge, which demonstrates that the photogenerated charge separation efficiency of 2D-TiO_2_ is superior to that of 1D-TiO_2_. To elucidate the charge migration characteristics and complex formation in the catalysts, electrochemical impedance spectroscopy (EIS) analysis was carried out. As illustrated in Figure 5b, the Nyquist plot diameter of 2D-TiO_2_ is smaller than that of 1D-TiO_2_, thereby directly demonstrating that 2D-TiO_2_ possesses a lower resistance. This phenomenon can be attributed to a more rapid transfer of photogenerated carriers and a significant inhibition of photogenerated carrier complexation [37,38,39]. The present findings are consistent with those reported in earlier studies [40,41,42]. In order to investigate the effect of the composites on the physical properties of the interface, static water contact angle tests were carried out on 1D-TiO_2_ and 2D-TiO_2_ (Figure 5c,d). The contact angle of 2D-TiO_2_ reaches 34.5°, while the contact angle of 1D-TiO_2_ is 43.5°, with an angular difference of about 10°. When the contact angle is less than 90°, the material is considered hydrophilic and wettable, and when the contact angle is greater than 90°, it is considered non-wettable. This indicates that either 2D-TiO_2_ or 1D-TiO_2_ is hydrophilic. For hydrophilic materials, it was determined that an increase in surface roughness was accompanied by an enhancement in surface hydrophilicity. This finding aligns with the conclusions drawn from the SEM analysis. In comparison, 2D-TiO_2_ exhibits superior hydrophilicity. The surface of 2D-TiO_2_ is more likely to adsorb water, which facilitates the contact between the surface of the catalyst and CO_2_, and results in the provision of more active sites. Furthermore, the reduction in contact angle facilitates increased interaction between the water molecules and the catalyst surface, thereby enhancing the diffusion of water molecules on the catalyst surface, accelerating the mass transfer process between the catalyst and the reactants, and consequently improving reaction efficiency. Finally, the small contact angle is more conducive to the provision of a constant proton source during the reaction process, which further proves the good photocatalytic performance of 2D-TiO_2_ [43,44].

**Figure 6:**
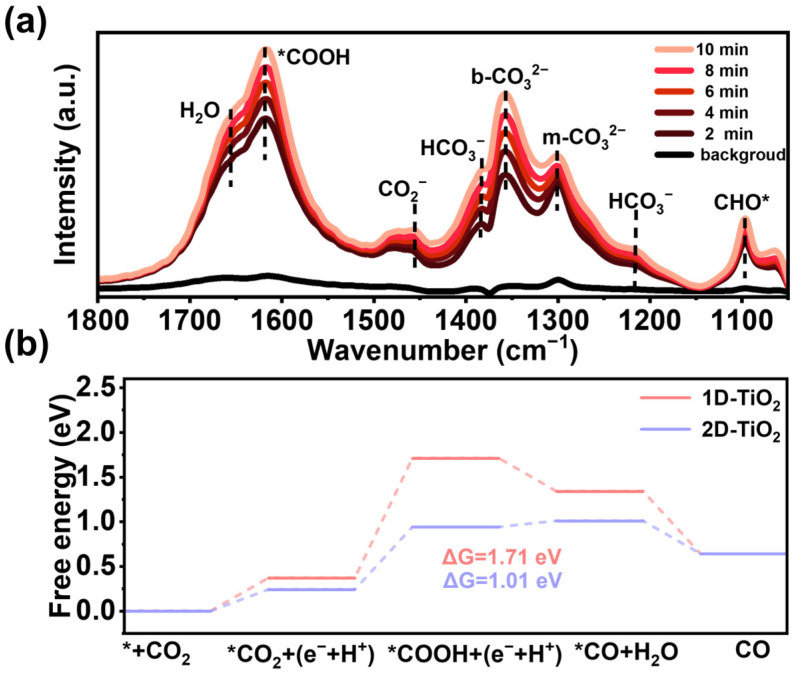
(**a**) In situ FT-IR spectra of 2D-TiO_2_ at different irradiation times. (**b**) Gibbs free energy calculations for the photocatalytic reduction of CO_2_ by 2D-TiO_2_ and 1D-TiO_2_. (* represents photocatalyst).

To gain a deeper understanding of the reaction mechanism involved in photocatalytic CO_2_ reduction, this study employed in situ FT-IR and Gibbs free energy to study the dynamic process of the photocatalytic CO_2_ reduction reaction. The samples were dried under vacuum at 100 °C for one hour prior to in situ FT-IR measurements. The objective of this procedure was to eradicate adsorbed interfering substances. After the introduction of high-purity CO_2_ into the photocatalytic reaction system, raw FT-IR spectra were initially collected in the absence of light to obtain raw blank values. After the irradiation of the sample, the blank values were subtracted to obtain FT-IR spectra. In situ FT-IR spectra were collected in the presence of light in 2 min intervals. The results demonstrated that no discernible absorption peaks were observed under dark conditions. However, under light conditions, the catalyst demonstrated absorption peaks corresponding to the intermediates (Figure 6a). These corresponded to H_2_O (1656 cm^−1^), *COOH (1619 cm^−1^), CO_2_^−^ (1453 cm^−1^), HCO_3_^−^ (1383 cm^−1^ and 1214 cm^−1^), b-CO_3_^2−^ (1357 cm^−1^), m-CO_3_^2−^ (1298 cm^−1^), and *CHO (1096 cm^−1^). It was evident that the absorption peaks underwent a substantial enhancement in conjunction with an increase in the duration of the light exposure [45,46]. The enhancement of the peaks for surface adsorbed water is assumed to be due to the successive adsorption decomposition of H_2_O, which provides a constant supply of H^+^ involved in the reaction [47]. The most significant increases were observed for *COOH, b-CO_3_^2−^ and m-CO_3_^2−^. These compounds are pivotal in the reduction of CO_2_ to CO by 2D-TiO_2_. Furthermore, the increase in peak intensity of *CHO (the crucial intermediate in CH_4_ formation) is negligible. These findings also support the conclusion that 2D-TiO_2_ promotes selective product generation during CO_2_ reduction. In consideration of the results obtained from in situ FT-IR, it can be posited that the most plausible pathway within the 2D-TiO_2_ photoreduction of the CO_2_ system may be as follows:

(1) $$\begin{array}{c} {\mathrm{CO}}_{2}+*\to *{\mathrm{CO}}_{2} \end{array}$$

(2) $$\begin{array}{c} {}*{\mathrm{CO}}_{2}+H^{+}+e^{-}\;\to \;*COOH \end{array}$$

(3) $$\begin{array}{c} \begin{array}{c} {}*COOH+H^{+}+e^{-}\;\to \;*CO+H_{2}O \end{array} \end{array}$$

(4) $$\begin{array}{c} {}*CO\;\to \;CO↑+\;* \end{array}$$

In these equations, the ‘*’ symbol indicates the adsorption state of the intermediate on the photocatalyst surface. On this basis, the effect of morphology on the Gibbs free energy (ΔG) during the whole reaction process was investigated by using DFT calculations. As demonstrated in Figure S7, the photocatalyst absorbs photons to generate photogenerated electron-hole pairs. The electrons migrate to the surface to reduce CO_2_, and the holes oxidize water or donors. The arrows indicate the migration of photogenerated carriers, CO_2_ adsorption, and product desorption. As demonstrated in Figure 6b, Figures S8 and S9, the adsorption model during the reaction can be observed to identify the adsorption of CO_2_ on the catalyst surface as a high-energy step and the formation of *CO and *COOH intermediates on 2D-TiO_2_ as a key step in CO_2_ reduction [48,49]. In the case of both 1D-TiO_2_ and 2D-TiO_2_ catalysts, the pivotal step is the adsorption of CO_2_. It has been demonstrated that changes in morphology notably impact the reaction energy barrier and the formation energy of key intermediates [50,51]. Hence, the thermodynamic energy barrier necessary for the reaction is reduced, resulting in a substantial increase in activity [52].

## 4. Conclusions

In summary, 2D-TiO_2_ and 1D-TiO_2_ were synthesized by a hydrothermal method, and various characterization tests were carried out on them. It was concluded that the photocatalytic performance of 2D-TiO_2_ was significantly better than that of 1D-TiO_2_. The conversion rate of CO_2_ to CO by 2D-TiO_2_ has been demonstrated to be up to 24.53 μmol g^−1^ h^−1^, exhibiting a 66% CO selectivity. The plate-like structure of the material under investigation provides a larger specific surface area, thereby promoting photoelectron transport and charge separation. DFT calculations further reveal the optimized electronic structure of the 2D-TiO_2_ surface. This lowers the free energy barrier of the rate-limiting step (*CO_2_ → *COOH) and enhances CO_2_ adsorption and activation, providing theoretical support for the experimental results. This study provides new data for the development of 2D-TiO_2_ catalysts with high photocatalytic CO_2_ reduction activity, which has great potential for application.

## Data Availability

The original contributions presented in this study are included in the article. Further inquiries can be directed at the corresponding author.

## Supplementary Materials

The following supporting information can be downloaded at: https://www.mdpi.com/article/10.3390/ma18174148/s1, Figure S1: The structural models of 2D-TiO_2_ and 1D-TiO_2_. Figure S2: The band structure of (a) 2D-TiO_2_ and (b) 1D-TiO_2_. Figure S3: XRD patterns of 2D-TiO_2_ and 1D-TiO_2_. Figure S4: FT-IR spectra of 2D-TiO_2_ and 1D-TiO_2_. Figure S5: Comparison of photocatalytic carbon dioxide reduction performance of 2D-TiO_2_ and P25. Figure S6: XRD patterns of 2D-TiO_2_ before and after the cycling photocatalytic experiment. Figure S7: Photocatalytic reduction of CO_2_ mechanism diagram. Figure S8: Structural models of Gibbs free energy calculations on 2D-TiO_2_. Figure S9: Structural models of Gibbs free energy calculations on 1D-TiO_2_. Table S1: Comparison of photocatalytic CO_2_ reduction performance with recently reported layered double hydroxide photocatalysts. References [53,54,55,56,57,58] are cited in the Supplementary Material.
