# Supplementary material for: Dimensional Engineering of 1D/2D Synergistic TiO2 Nanostructures for High-Efficiency Photocatalytic CO2 Reduction
Source: Materials (Basel). 2025 Sep 4;18(17):4148. doi: 10.3390/ma18174148 (PMC12430599; doi:10.3390/ma18174148)
Supplement: Supplementary file 1 [file materials-18-04148-s001.zip › materials-3839953-supplementary.pdf]

materials

Supplementary Information

Preparation of a Novel Carbon Nano Coating on Carbon Fiber Surface Based on Plasma Electrolysis Effect

Xin He [1], Qian Zhou [1], Maoyuan Li [2], Dongqin Li [1], Chiyuhao Huang [1], Xiaolin Wei [1] and Weiwei Chen [1,*]

[1] Department of Materials Science and Engineering, Beijing Institute of Technology, Beijing 100081, China
[2] Beijing System Design Institute of Electro-Mechanic Engineering, Beijing 100854, China
* Correspondence: wwchen@bit.edu.cn

Characterization of the photocatalysts

The X-ray diffraction (XRD) patterns of the powders were recorded using a Bruker D8 diffractometer with Cu Kα radiation (λ = 1.5406 Å) at a scan rate of 7° min^{-1}. The electron micrographs of the samples were analyzed using a field emission scanning electron microscope (FE-SEM, S4800II) with an accelerating voltage of 5.0 kV. High-resolution TEM (HRTEM) images and elemental mapping of the samples were obtained using a Tecnai 12 with an accelerating voltage of 120 kV. The optical properties of the samples were determined using a Cary 5000 UV/VIS/NIR absorption spectrometer. In situ Fourier transform infrared (FT-IR) spectra were obtained by the Thermo Scientific Nicolet iS50. The specific area was measured by the Brunauer–Emmett–Teller (BET) method using the N_2 adsorption–desorption isotherm (ASAP 2460, Micromeritics Instrument). The contact angle of water droplets on the corresponding samples was measured with the JCY-2 contact angle tester (measuring range 0–180°).

Photoelectrochemical Test

The photocurrent and impedance were measured using a DH97000C electrochemical system with a standard three-electrode system. A total of 5 mg of sample was added to a solution containing 50 μL of Nafion and 950 μL of ethanol, and the solution was sonicated to mix it well. Then 50 μL of the suspension was pipetted and dropped onto indium oxide (ITO)-coated glass and dried over an area of 1 cm*1 cm. For the experiments, the prepared ITO was used as the working electrode, Pt wire was used as the counter electrode, and Ag/AgCl was used as the reference electrode. A 0.2 M Na_2SO_4 solution was used as the electrolyte solution, and a 300 W Xe lamp was used as the light source.

Photocatalytic CO_2 reduction measurement

Based on an online system, the photocatalytic CO_2 reduction reaction was carried out in a quartz glass reactor (μGAS1000, Beijing Perfectlight Technology Co., Ltd., China). The experimental steps were as follows: 6 mL of acetonitrile, 4 mL of H_2O, 2 mL of triethanolamine, and 10 mg of catalyst were taken to formulate the hole sacrificial regent and stirred with a magnetic stirrer. Then irradiation was carried out with a 300 W xenon lamp at a temperature of 10 °C and a pressure of 75 kPa. During irradiation, 1 mL of gas was withdrawn from the reaction cell every 60 min, and the gas was characterized using a gas chromatograph (GC 9720, Ar carrier) equipped with a thermal conductivity detector (TCD) and a hydrogen flame ionization detector (FID). Mainly CO and CH_4 gases were detected. The yield was quantified according to the correction curve. The selectivity of

 https://doi.org/10.3390/ma18174148

CH_4 was calculated as CH_4 (%) = [8ν(CH_4)]/[2ν(CO) + 8ν(CH_4) + 2ν(H_2)] × 100%, where ν(CO), ν(CH_4), and ν(H_2) stand for the yielding rates of CO, CH_4, and H_2, respectively.

Computational methods

In this work, density functional theory (DFT) calculations were performed for structural optimization implemented in the Vienna ab initio Simulation Package (VASP). The generalized gradient approximation (GGA) of the PBE function, the all-electronic plane-wave basis set (energy cut off: 520 eV), a maximum force tolerance of 0.05 eV/Å, and the projector-augmented wave (PAW) method were used to describe the exchange-correlation energy. In order to obtain the exact density of electronic states, a 3×3×1 Monkhorst–Pack grid was used for Brillouin zone integration during the iterative process. Ion relaxation was performed under conventional energy (1×10^{-5} eV) and force (0.02 eV/Å) convergence criteria. By testing all possible configurations of CO_2*, COOH* and CO* adsorbed on the possible active sites on the CdS surface, their ground state structures were determined and the lowest energy configuration was found. The free energy of adsorbed and non-adsorbed gas phase molecules is calculated as:

$$\Delta G = E_{total} - E_{slab} - E_{mol} + \Delta E_{ZPE} - T\Delta S \quad (s1)$$

where E_{total} is the total energy of the adsorbed state, E_{slab} is the pure surface energy, E_{mol} is the adsorbed molecular energy, ΔE_{ZPE} is the zero-point energy change, and ΔS is the entropy change.

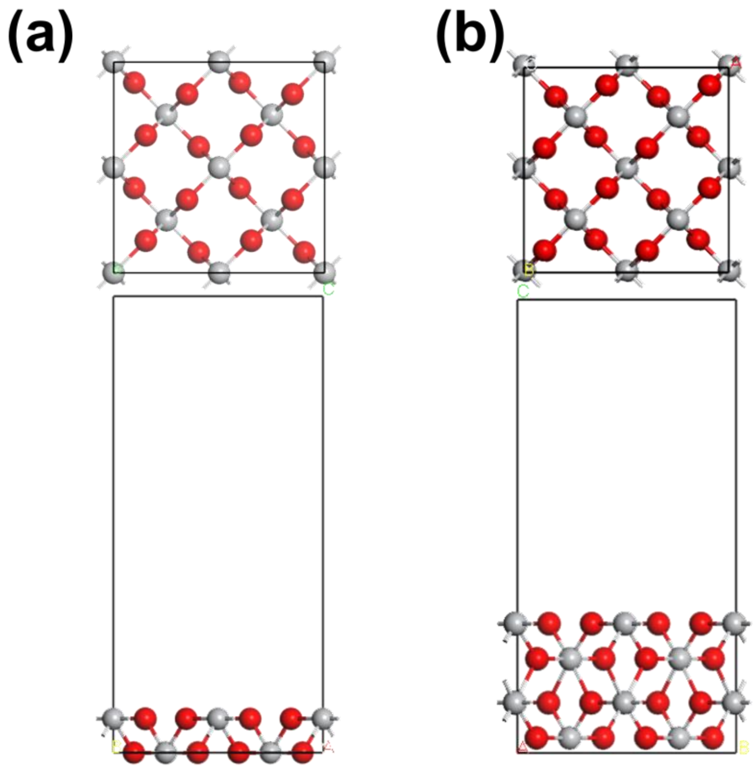

Figure S1. The structural models of 2D-TiO_2 and 1D-TiO_2.

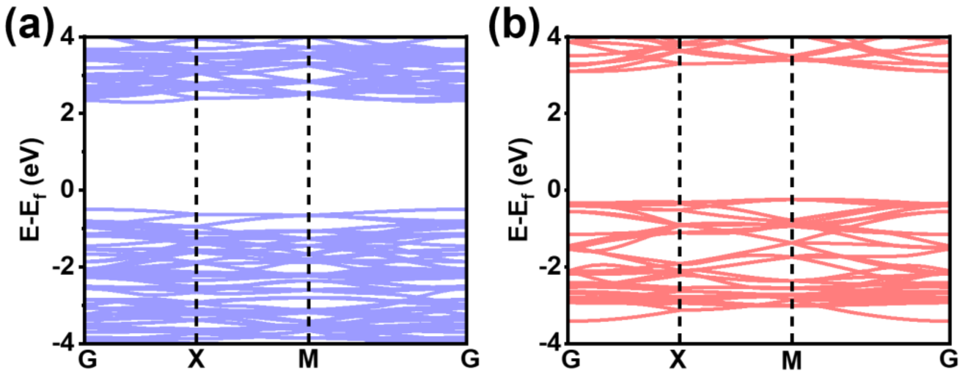

Figure S2. The band structure of (**a**) 2D-TiO_2 and (**b**) 1D-TiO_2.

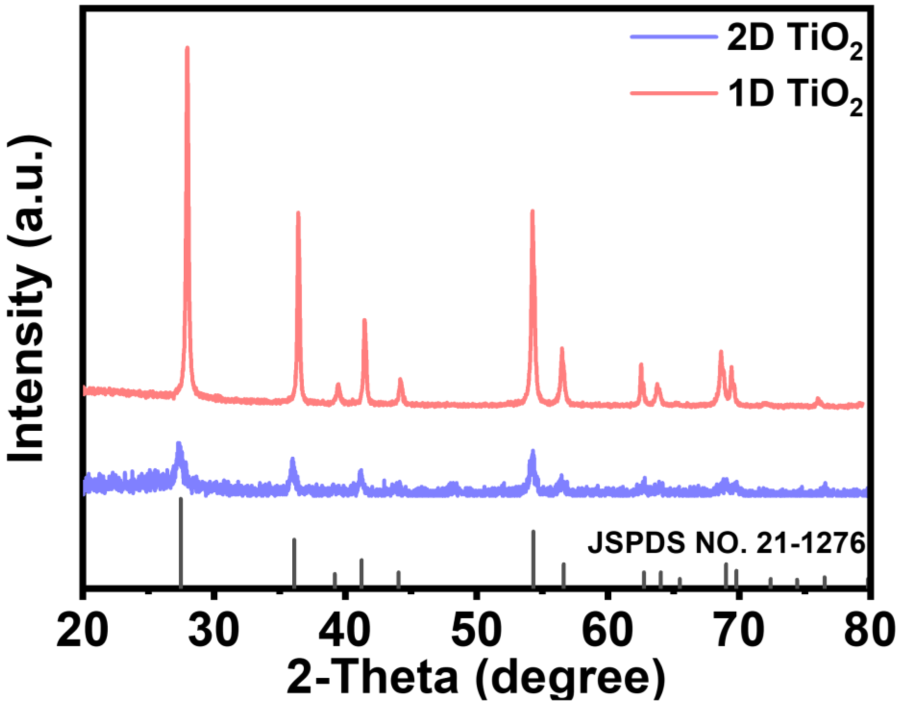

Figure S3. XRD patterns of 2D-TiO_2 and 1D-TiO_2.

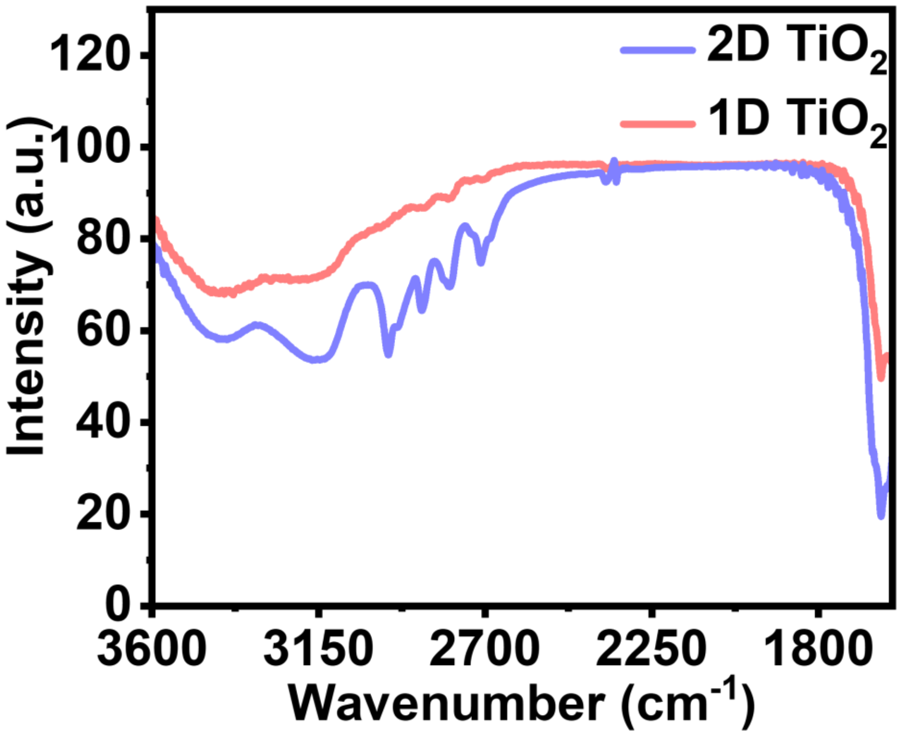

Figure S4. FT-IR spectra of 2D-TiO_2 and 1D-TiO_2.

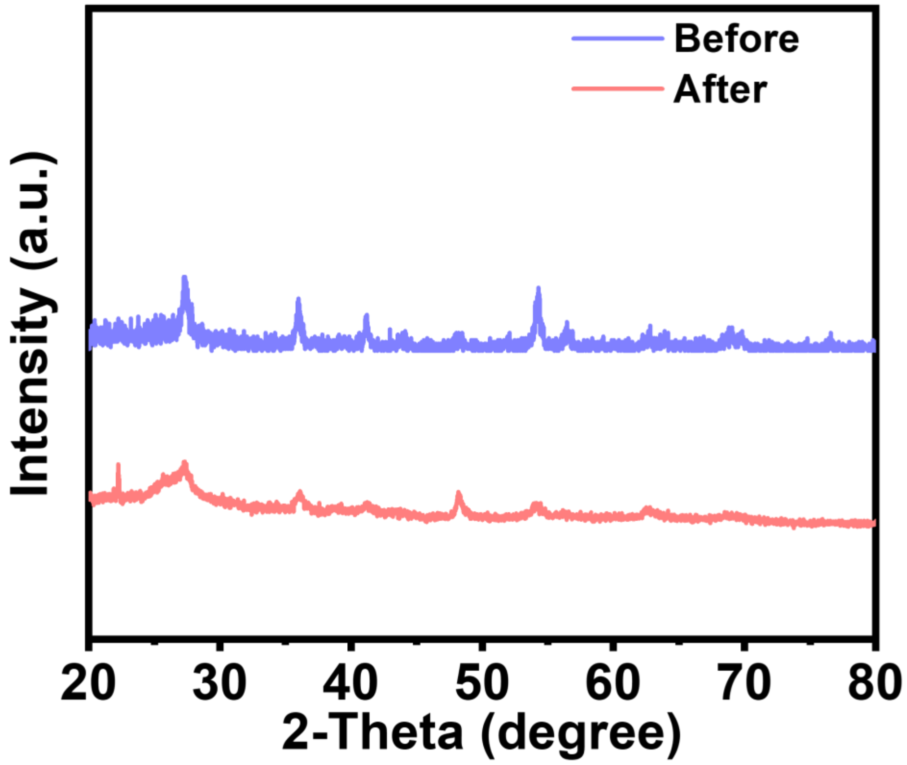

Figure S5. XRD patterns of 2D-TiO_2 before and after the cycling photocatalytic experiment.

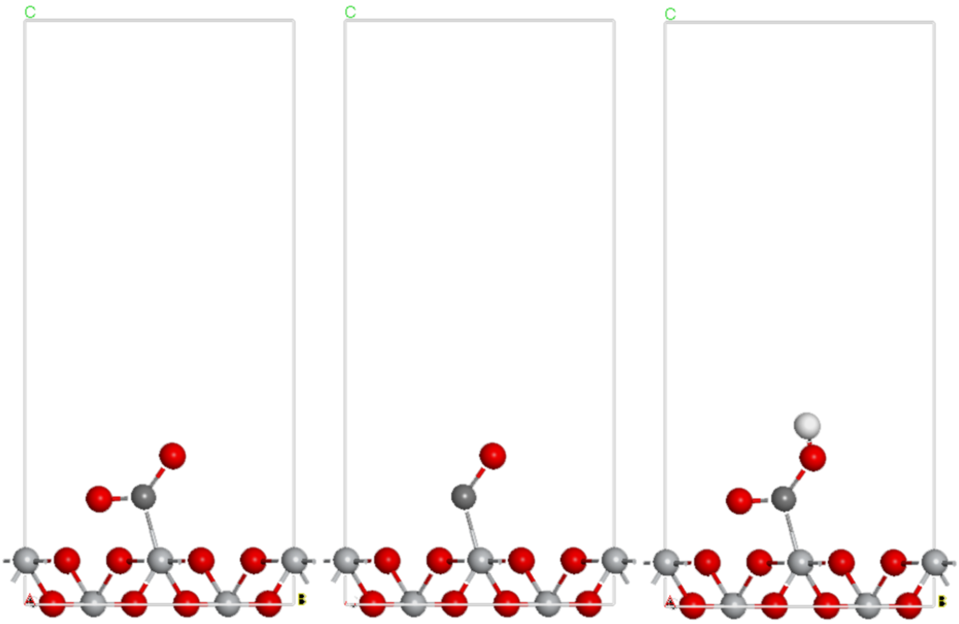

Figure S6. Structural models of Gibbs free energy calculations on 2D-TiO_2.

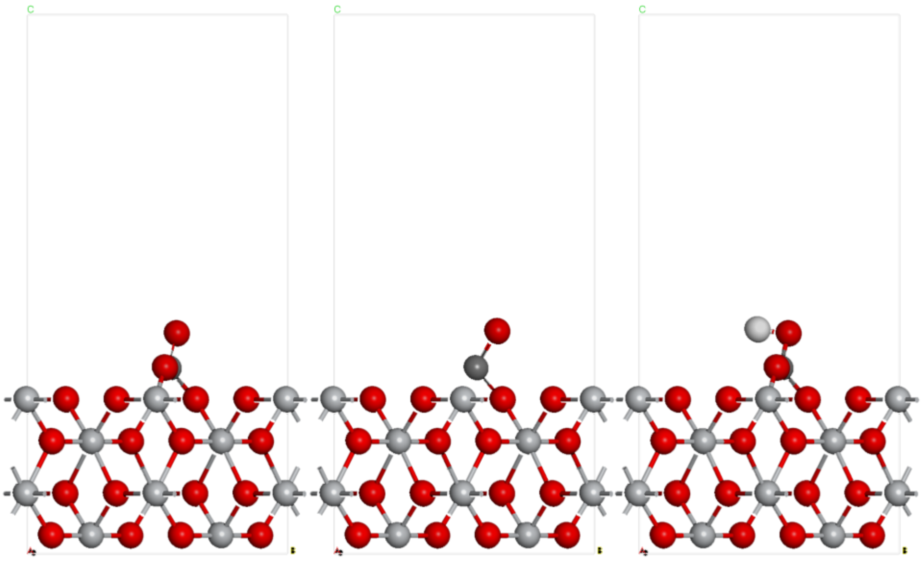

Figure S7. Structural models of Gibbs free energy calculations on 1D-TiO_2.

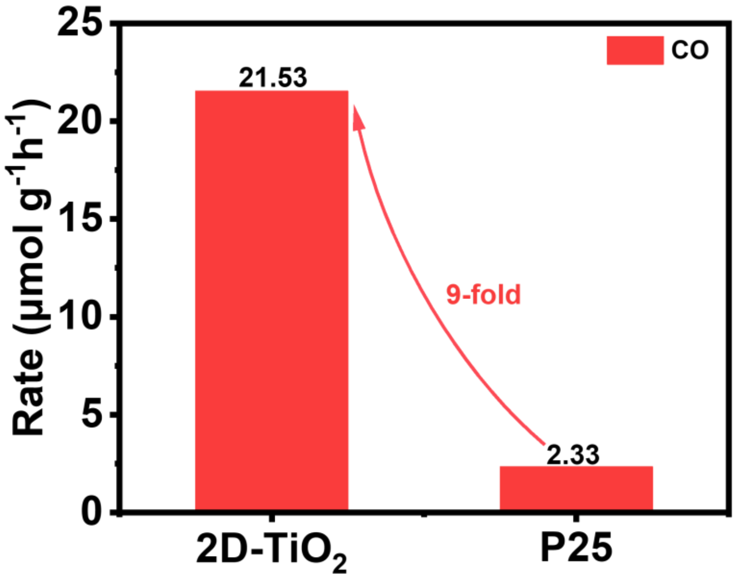

Figure S8. Comparison of photocatalytic carbon dioxide reduction performance of 2D-TiO_2 and P25 [1].

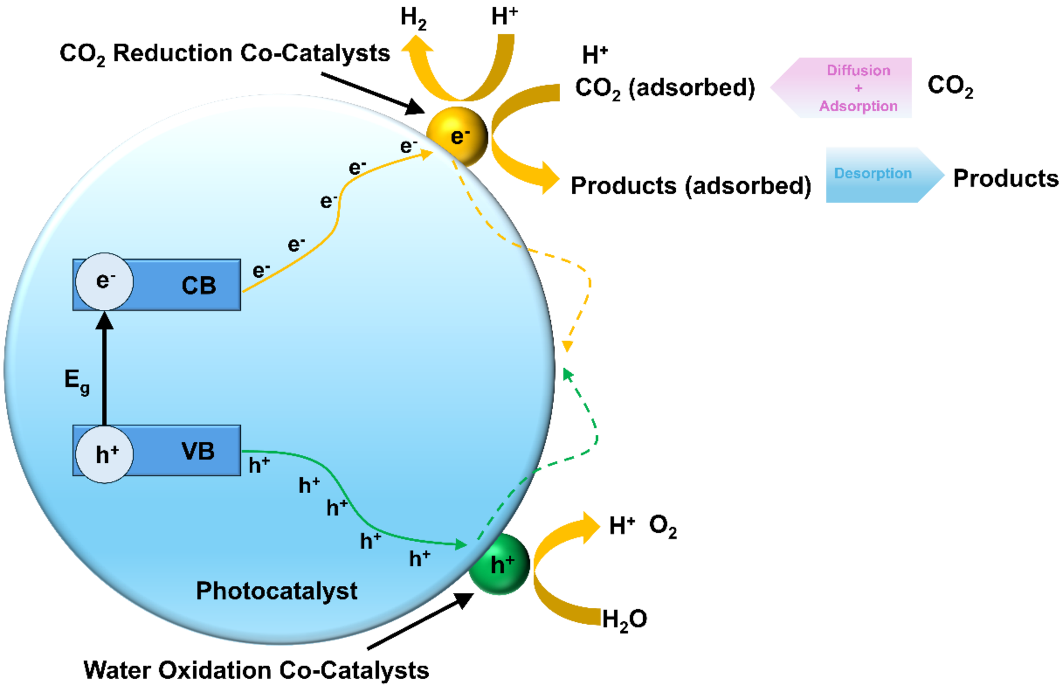

Figure S9. Photocatalytic reduction of CO_2 mechanism diagram.

Table S1. Comparison of photocatalytic CO_2 reduction performance with recently reported layered double hydroxide photocatalysts.

Photocatalyst	Reaction medium	Main products (μmol g^{-1} h^{-1})	Reference
2D-TiO_2	300 W Xe-lamp, TEOA, H_2O, MeCN, 10 mg catalyst	21.53 (CO)	This work
95% (100) facets of TiO_2 nanosheets	UV, H_2O, 40 mg catalyst	5.8 (CO)	[2]

TiO_2-Bi	300 W Xe-lamp, TEOA, H_2O, MeCN, 10 mg catalyst	6.38 (CO)	[3]
Mixed-phase anatase nanosheets/brookite nanorods TiO_2	300 W Xe-lamp, H_2O, 10 mg catalyst	~2 (CO)	[4]
Ultrathin TiO_2 nanosheet	300 W Xe-lamp, TEOA, H_2O, MeCN, 50 mg catalyst	6.3 (CO)	[5]
TiO_2-x	300 W Xe-lamp, H_2O, 1 mg catalyst	0.411 (CO)	[6]
Cu/ TiO_2	Solar light, H_2O, 5 mg catalyst	3.2 (CO)	[7]

References

1. KE, Y.-H.; ZENG, M.; JIANG, H.; XIONG, C.-R., Photocatalytic Reduction of Carbon Dioxide to Methanol over N-doped TiO_2 Nanofibers under Visible Irradiation. *J. Inorg. Mater.* **2018,** 33, (8), 839-844.
2. Xu, H.; Ouyang, S.; Li, P.; Kako, T.; Ye, J., High-active anatase TiO_2 nanosheets exposed with 95% {100} facets toward efficient H_2 evolution and CO_2 photoreduction. *ACS Appl Mater Interfaces.* **2013,** 5, (4), 1348-54.
3. Li, X.; Bi, W.; Wang, Z.; Zhu, W.; Chu, W.; Wu, C.; Xie, Y., Surface-adsorbed ions on TiO_2 nanosheets for selective photocatalytic CO_2 reduction. *Nano Res.* **2018,** 11, (6), 3362-3370.
4. Ioannidou, T.; Anagnostopoulou, M.; Vasiliadou, I. A.; Marchal, C.; Alexandridou, E.-O.; Keller, V.; Christoforidis, K. C., Mixed phase anatase nanosheets/brookite nanorods TiO_2 photocatalysts for enhanced gas phase CO_2 photoreduction and H_2 production. *J. Environ. Chem. Eng.* **2024,** 12, (1), 111644.
5. Wang, Z.-W.; Wan, Q.; Shi, Y.-Z.; Wang, H.; Kang, Y.-Y.; Zhu, S.-Y.; Lin, S.; Wu, L., Selective photocatalytic reduction CO_2 to CH_4 on ultrathin TiO_2 nanosheet via coordination activation. *Appl. Catal. B Environ.* **2021,** 288, 120000.
6. Dong, L.; Xiong, Z.; Zhou, Y.; Zhao, J.; Li, Y.; Wang, J.; Chen, X.; Zhao, Y.; Zhang, J., Photocatalytic CO_2 reduction over postcalcinated atomically thin TiO_2 nanosheets: Residual carbon removal and structure transformation. *J. CO2 Util.* **2020,** 41, 101262.
7. Jiang, Z.; Sun, W.; Miao, W.; Yuan, Z.; Yang, G.; Kong, F.; Yan, T.; Chen, J.; Huang, B.; An, C.; Ozin, G. A., Living Atomically Dispersed Cu Ultrathin TiO_2 Nanosheet CO2 Reduction Photocatalyst. *Adv. Sci.* **2019,** 6, (15), 1900289.
